# Rosuvastatin protects against oxLDL-induced endothelial cell oxidative stress and attenuates atherosclerotic plaque formation in *ApoE*^*-/-*^ mice through the NF-κB pathway

**DOI:** 10.1371/journal.pone.0339967

**Published:** 2026-02-20

**Authors:** Yichen Wu, Jiaqi Ke, Jiaxin Lv, Chaowei Cao, Chunxian Zhou, Lili Wu, Qiyang Zhou

**Affiliations:** 1 Interventional Medicine Department, Suzhou Ninth Hospital Affiliated to Soochow University, Suzhou, Jiangsu, China; 2 Cyrus Tang Medical Institute, Soochow University, Suzhou, Jiangsu, China; University of Manitoba, CANADA

## Abstract

Cardiovascular disease is one of the diseases with the highest global incidence and mortality rates, and atherosclerosis is its basic cause. Endothelial dysfunction induced by risk factors such as lipid oxidation or inflammatory stimulation is a critical stage in the development of atherosclerosis, with endothelial oxidative stress and apoptosis serving as important pathological bases. Rosuvastatin influences the occurrence of atherosclerosis by regulating lipid levels. In this study, we investigated the effects of rosuvastatin on ox-LDL-induced endothelial cell injury and atherosclerosis. The results showed that intragastric administration of rosuvastatin inhibited high-fat diet (HFD)-induced changes in the aortic plaque area and aortic root lipid deposition in mice. In addition, rosuvastatin reduced mouse body weight and decreased the plasma levels of low-density lipoprotein (LDL) and total cholesterol (TC). The in vitro results demonstrated that rosuvastatin suppressed ox-LDL-induced endothelial oxidative stress, promoted the expression of nitric oxide (NO) and endothelial nitric oxide synthase (eNOS), and reduced intracellular reactive oxygen species (ROS) production. Additionally, rosuvastatin protected against ox-LDL-induced endothelial apoptosis by increasing Bcl-2 expression and decreasing Bax expression. Mechanistically, rosuvastatin inhibited the activation of the NF-κB signaling pathway induced by ox-LDL and suppressed the phosphorylation of P65, thereby reducing the expression of molecules related to oxidative stress and apoptosis. In conclusion, this study suggests that rosuvastatin may attenuate atherosclerosis by inhibiting endothelial oxidative stress and apoptosis, which provides a theoretical basis for the prevention and treatment of atherosclerosis.

## Introduction

Atherosclerosis (AS) is the most common pathological basis of cardiovascular disease (CVD) and is driven by many factors, such as dyslipidemia, diabetes, hypertension, and hemodynamic disorders [1]. Endothelial dysfunction is a typical early manifestation of atherogenesis, as well as the basic pathogenesis of multiple CVDs, including hypertension, coronary disease, angina pectoris and cardiac failure. Endothelial cell dysfunction is characterized by the loss of endothelial barrier integrity, increased secretion of inflammatory factors, and an imbalance in oxidative stress [2]. Lipid metabolism disorders are among the most important causes of impaired endothelial cell function and result in a series of oxidative stress reactions. Oxidized low-density lipoprotein (ox-LDL), a key pathogenic factor in atherosclerosis, triggers a series of pathological processes by damaging endothelial cells. Ox-LDL can induce endothelial cells to express adhesion molecules, such as VCAM-1 and ICAM-1 [3], promote the adhesion and infiltration of monocytes, and increase the generation of reactive oxygen species (ROS), leading to increased endothelial permeability and impaired endothelial cell function [4], which play a role in proinflammatory and immune stimulatory effects, thereby promoting the occurrence and development of AS. Endothelial cell dysfunction is positively correlated with the progression of atherosclerosis, and the degree of damage to endothelial cells directly affects the stability of plaques and clinical outcomes [5]. For example, in the *ApoE*^*⁻/⁻*^ mouse model, ox-LDL-induced endothelial oxidative stress can accelerate plaque formation, whereas inhibiting ox-LDL uptake significantly reduces the lesion burden [6–8]. Therefore, understanding the mechanism of ox-LDL in endothelial cell dysfunction and finding drugs to regulate endothelial cell oxidative stress are crucial for the prevention and treatment of atherosclerosis.

Oxidative stress is a key mechanism linking endothelial injury to atherosclerosis. Excessive production of reactive oxygen species (ROS) in endothelial cells can directly activate inflammatory signaling pathways such as the NF-κB and MAPK pathways, leading to increased expression of proinflammatory factors (e.g., IL-1β and TNF-α) and matrix metalloproteinases, which promote plaque erosion and rupture [9]. Studies have confirmed that ox-LDL disrupts the normal function of endothelial nitric oxide synthase (eNOS) through oxidative stress and inflammatory signals, triggering eNOS uncoupling, where the enzyme shifts from producing nitric oxide (NO) to generating superoxide anions [10]. The superoxide anions produced by this uncoupling synergize with ox-LDL to form a positive feedback loop of oxidation and inflammation. Additionally, NO deficiency directly disrupts endothelial barrier function and activates inflammatory responses, which manifest as impaired endothelium-dependent vasodilation in atherosclerotic animal models [11]. For example, after intragastric administration of ox-LDL in *ApoE*^*⁻/⁻*^ mice, the diastolic response of the aorta to acetylcholine was significantly weakened [12,13]. Furthermore, studies have shown that in human umbilical vein endothelial cells (HUVECs) treated with ox-LDL, eNOS phosphorylation levels decrease, NO release decreases, and the proportion of Annexin V-positive cells increases, indicating a significant increase in the apoptosis rate [10]. Moreover, ox-LDL not only inhibits eNOS activity but also reduces eNOS protein expression through transcriptional and posttranslational mechanisms. In vivo experiments have demonstrated that after the intraperitoneal injection of ox-LDL in *ApoE*^*⁻/⁻*^ mice, eNOS expression in the aortic endothelium is significantly downregulated, accompanied by increased expression of the adhesion molecule VCAM-1 and increased monocyte infiltration [14].

Rosuvastatin, an inhibitor of 3-hydroxy-3-methylglutaryl-coenzyme A reductase, is the current paradigm for lipid management and is used to ameliorate abnormal lipid levels to improve lipid metabolism [15]. Therefore, in-depth research into its mechanisms of action may help improve therapeutic efficacy. The lipid-lowering effect of rosuvastatin is critical for its ability to regulate atherosclerosis. By inhibiting HMG-CoA reductase, a key enzyme in hepatic cholesterol synthesis, rosuvastatin reduces endogenous cholesterol production, lowers blood low-density lipoprotein cholesterol (LDL-C) levels, decreases lipid deposition in the vascular wall, and fundamentally weakens the pathological basis of atherosclerosis [16]. Additionally, rosuvastatin has anti-inflammatory effects, which represent another important functional mechanism involved in its regulation of atherosclerosis. It can reduce the levels of inflammatory markers such as high-sensitivity C-reactive protein (hs-CRP), alleviate inflammatory responses in the vascular wall, stabilize plaques, and reduce the risk of cardiovascular events [17]. Relevant clinical studies have shown that rosuvastatin can reduce atherosclerosis and symptoms of stable angina pectoris, as well as decrease the size of vulnerable coronary artery plaques [18]. A previous study demonstrated that rosuvastatin was more effective at lowering LDL-C than was atorvastatin and that it decreased plaque volume and vascular volume in vulnerable coronary artery plaques in patients with AS and stabilized angina pectoris, which are closely associated with endothelial dysfunction [19]. Studies have shown that rosuvastatin can inhibit endothelial inflammatory responses and reduce monocyte adhesion to endothelial cells [20]. However, whether rosuvastatin can influence endothelial oxidative stress and its underlying mechanisms remain unclear. This study investigated the effects of rosuvastatin on the viability and function of ox-LDL-treated HUVECs and validated the protective effects of rosuvastatin against atherosclerosis in *ApoE ⁻ / ⁻* mice, providing a theoretical basis for exploring the clinical applications of statin drugs.

## Materials and methods

### Animals and reagents

*ApoE*^*-/-*^ mice (C57BL/6J background) were purchased from Jackson Laboratories (Bar Harbor, USA) and housed on a light/dark (12 hour/12 hour) cycle at 24°C in a specific pathogen-free facility, and all experiments were approved by the Institutional Animal Care and Use Committee of Soochow University (20211231). Eight-week-old male mice were fed a high-fat diet (HFD) (0.15% cholesterol and 21% fat without added cholate; Harlan Teklad, 88137, USA) for 6 weeks, and the mice were randomly divided into two groups (n = 6 in each group). The model group was given an equal volume of PBS by gavage, while the RSV treatment group was given a dose of 5 mg/kg by gavage every two days. Both groups of mice were fed a high-fat diet for 6 weeks. The mice were anesthetized via an intraperitoneal injection of 1% pentobarbital (7 μL/g), and the depth of anesthesia was monitored by monitoring the absence of the pedal reflex. At the end of all the experiments, the mice were euthanized by CO_2_ inhalation at a rate of 1.9–4.4 L/min for 20 min, followed by cervical dislocation to minimize animal suffering. Rosuvastatin (Cat# HY-17504) was purchased from MedChemExpress (New Jersey, USA). ox-LDL (Cat# YB-002) was purchased from Yiyuan Biotechnologies (Guangzhou, China). A nitric oxide detection kit (Cat# S0021S) and a reactive oxygen species (ROS) detection kit (Cat# S0033S) were purchased from Beyotime (Shanghai, China). The primary antibodies used in this study included rabbit anti-Bax (Cat# AF0120, Affinity Biosciences, USA), rabbit anti-Bcl2 (Cat# AF6139, Affinity Biosciences, USA), rabbit anti-IkBα (Cat# 4812, CST, USA), rabbit anti-phospho-IκBα (Ser32) (Cat# 2859, CST, USA), rabbit anti-NF-κB p65 (Cat# 8242, CST, USA), rabbit anti-phospho-NF-κB p65 (Ser536) (Cat# 3033, CST, USA), rabbit anti-actin (Cat# AC026, ABclonal, China), rabbit anti-LC3 (Cat# 81004–1-RR, Proteintech, China), mouse anti-P62 (Cat# 66184–1-Ig, Proteintech, China), and mouse anti-tubulin (Cat# S0B0645, STARTER, China) antibodies. The secondary antibodies used were HRP-conjugated AffiniPure goat anti-rabbit IgG (H + L) (1: 5000, SA00001−2, Proteintech) and HRP-conjugated AffiniPure goat anti-mouse IgG (H + L) (1: 5000, SA00001−1, Proteintech).

### Analysis of atherosclerotic lesions

Atherosclerotic plaques were quantified in en face preparations stained with Sudan IV. Aortic images encompassing the entire aorta (arch, thorax, and abdomen) were taken via an Olympus SZX16 Telescope (Olympus, Japan) and analyzed via Olympus cellSens Standard software (Japan). For the aortic roots, the hearts were cut at 10 µm and stained with Oil Red O (Cat# D027, NJJC, China), followed by counterstaining with hematoxylin.

### Cell culture

Primary human umbilical vein endothelial cells (HUVECs) (Cat#PCS-100–010, ATCC, Maryland, USA) were maintained in vascular cell basal medium (Cat#PCS-100–030, ATCC, Maryland, USA) containing ascorbic acid (Cat#PCS-999–006, ATCC, Maryland, USA), FBS (Cat#PCS-999–010, ATCC, Maryland, USA), rhEGF (Cat#PCS-999–018, ATCC, Maryland, USA), heparin sulfate (Cat#PCS-999–011, ATCC, Maryland, USA), L-glutamine (Cat#PCS-999–017, ATCC, Maryland, USA), rhVEGF (Cat#PCS-999–024, ATCC, Maryland, USA), rhFGF-b (Cat#PCS-999–020, ATCC, Maryland, USA), rhIGF-1 (Cat#PCS-999–021, ATCC, Maryland, USA), and hydrocortisone (Cat#PCS-999–014, ATCC, Maryland, USA) at 37°C in an atmosphere containing 5% CO_2_.

### Cell activity assay and NO detection

The log-phase growing cells were seeded into 96-well plates at a density of 5,000 cells per well in 100 µL of medium and cultured for 24 hours. To evaluate the effects of ox-LDL and rosuvastatin on endothelial cell proliferative activity, endothelial cells were incubated with different concentrations of rosuvastatin (0.1, 1, 5, and 10 µM) or ox-LDL (50, 100, and 200 µg/mL) for 24 hours. Additionally, to investigate the effect of rosuvastatin on ox-LDL-induced endothelial cell injury, HUVECs were pretreated with different concentrations of rosuvastatin for 2 hours, followed by coincubation with 200 µg/mL ox-LDL or control solvent for 24 hours. Subsequently, 10 µL of CCK8 solution was added to each well, and the plates were incubated at 37°C for 2 hours in a 5% CO₂ incubator. The absorbance was then measured at 450 nm with a microplate reader (SpectraMax Plus384; Molecular Devices, LLC, Sunnyvale, CA, USA). The cell activity was normalized to that of the control group after the absorbance value of the blank group was subtracted from that of each well. For NO detection, the cell supernatant from the treated 96-well plates was transferred to new 96-well plates according to the kit manufacturer’s instructions. A standard curve was prepared using standard products, Griess reagent was added, and the plates were incubated at 37°C for 5 minutes in a 5% CO₂ incubator. The absorbance was measured at 540 nm, and the NO content was calculated on the basis of the standard curve.

### Detection of reactive oxygen species (ROS)

After the cells in the 96-well plate adhered to the wall, they were treated with different concentrations of rosuvastatin for 2 hours, followed by incubation with 200 µg/mL ox-LDL for 24 hours. The positive control group was set up simultaneously. The next day, in situ probes were loaded and incubated at 37°C for 20 minutes in a 5% CO₂ incubator. The fluorescence intensity was detected with a fluorescence microplate reader at an excitation wavelength of 488 nm and an emission wavelength of 525 nm. In addition, the cells in the 96-well plate were photographed via fluorescence microscopy (Olympus, Japan), and the green fluorescence intensity in the central field of view of each well was statistically analyzed using ImageJ software. The MFI of the control group was set as 100% to calculate the relative fluorescence intensity of each group.

### Flow cytometry analysis

After the cells in the 6-well plate had adhered to the wall, they were treated with different concentrations of rosuvastatin for 2 hours, followed by incubation with 200 µg/mL ox-LDL for 24 hours. The next day, the cell supernatant and adherent cells were collected and stained in the dark at 4°C for 15 minutes using an Annexin V-APC/PI Apoptosis Kit (FITC and PerCP-Cy5.5, Elabscience, E-CK-A217). All the experiments were performed under light-avoidance conditions. The final cells were analyzed by data acquisition using the Beckman CytoFLEX and FlowJo 10 software.

### Immunofluorescence staining

HUVECs treated with ox-LDL with or without rosuvastatin were fixed and incubated with a rabbit anti-Ki67 primary antibody (Cat# 28074–1-AP, Proteintech, China) or a rabbit anti-Bax antibody (Cat# AF0120, Affinity Biosciences, USA), and species-matched rabbit IgG was used as a negative control. Following primary antibody incubation, the cells were treated with Alexa Fluor 568-conjugated donkey anti-rabbit IgG (ab175470, Abcam, UK) or Alexa Fluor 647-conjugated donkey anti-rabbit IgG (ab150075, Abcam, UK) as the secondary antibody. The mitochondria were visualized via the use of a Mito-Tracker Red CMXRos kit (C1035, Beyotime, China). Nuclei were counterstained with DAPI, and coverslips were mounted with antifade mounting medium. Fluorescence images were acquired via a confocal laser scanning microscope (Olympus, Japan). For the quantification of Ki67-positive cells, 5 fields of view were randomly selected from each coverslip, the number of Ki67-positive cells was counted, and the mean value was taken as the representative data for that coverslip.

### Real-time quantitative PCR (RT‒qPCR)

Total RNA from HUVECs was extracted via an RNA simple Total RNA Kit (DP419, TIANGEN, China). Total RNA was reverse transcribed into cDNA using PrimeScript RT Master Mix (TAKARA, Japan). qPCR was performed in triplicate in 20 μL of the brilliant SYBR green PCR master mixture (4913914, Roche, Switzerland) in a real-time PCR system (LightCycler 480, Roche, Switzerland). The mRNA expression levels were normalized to the glyceraldehyde-3-phosphate dehydrogenase (GAPDH) level and are presented as relative fold changes via the 2-ΔΔCT method [21]. The sequences of the primers used were as follows: from 5’ to 3’. The eNOS forward primer (F) was TGATGGCGAAGCGAGTGAAG, and the reverse primer (R) was ACTCATCCATACACAGGACCC. Bcl-2-F is GGTGGGGTCATGTGTGTGG, and Bcl-2-R is CGGTTCAGGTACTCAGTCATCC. Bax-F is CCCGAGAGGTCTTTTTCCGAG, and Bax-R is CCAGCCCATGATGGTTCTGAT. GAPDH-F is ACAACTTTGGTATCGTGGAAGG, and GAPDH-R is GCCATCACGCCACAGTTTC.

### Preparation of cell lysates and immunoblotting

The cells were lysed on ice in RIPA lysis buffer (1% Triton X-100, 1% deoxycholate, 0.1% SDS, 10 mM Tris and 150 mM NaCl) containing a protease and phosphatase inhibitor cocktail (Santa Cruz Biotechnology Inc., Heidelberg, Germany). The cell lysates were sonicated via ultrasonication (Sonics, China, Cat#VCX130) and centrifuged at 14,000 × g for 5 min at 4°C. The protein concentration of the supernatant was measured via a BCA protein assay kit (Beyotime, China). The reduced proteins (30 μg) in 4 × sample buffer (Invitrogen) containing β-2-mercaptoethanol were heated at 95°C for 5 min before loading. Protein samples were separated on a 10% gel and transferred to PVDF membranes. The membranes were blocked with 5% nonfat dry milk (Bio-Rad, Calif, USA) in TBS-T and incubated with primary antibody overnight at 4°C. Primary antibody binding was detected with a secondary antibody conjugated to HRP (1:5,000) for 1 h at room temperature. Bands were visualized via enhanced chemiluminescence (ECL). Densitometric analysis was performed via ImageJ software (NIH) to quantify protein expression levels, with β-actin or β-Tubulin used as internal controls.

### Measurement of lipid levels in serum

Serum levels of TG (triglycerides, Cat# A110-1–1), TC (total cholesterol, Cat# A111-1–1), HDL-C (high-density lipoprotein, Cat# A112-1–1) and LDL-C (low-density lipoprotein, Cat# A113-1–1) in overnight fasted mice were examined via assay kits from Nanjing Jiancheng Bioengineering Institute according to the manufacturer’s instructions. We collected the serum and used Shanghai Majorbio Bio-Pharm Technology Co., Ltd., to conduct nontargeted metabolomics mass spectrometry analysis of the plasma metabolites.

### Bioinformatics analysis

Transcriptome sequencing data of endothelial cells stimulated with ox-LDL were downloaded from the GEO database [22] (accession number GSE206927). Bioinformatics analysis of the sequencing data was conducted. The DAVID database platform was used to carry out gene function annotation research, including Gene Ontology (GO) functional classification and KEGG pathway enrichment analysis. The results are presented via the plotting package of R Studio.

### Statistics

The data are presented as the means ± S.E.M.s (standard error of the mean) and were analyzed via GraphPad Prism version 8. The animal sample size was analyzed by G.Power 3.1 software. All the quantitative data were tested for normality using the Shapiro-Wilk test (P > 0.05) to confirm their normal distribution. For comparisons between two groups with a normal distribution, unpaired Student’s *t t*ests were performed. One-way ANOVA followed by Tukey’s or Dunnett’s multiple comparisons test or two-way ANOVA with Tukey’s multiple comparisons test was used to compare data from three or more groups with a normal distribution. *P* < 0.05 was considered statistically significant.

## Results

### Rosuvastatin inhibits the formation of atherosclerotic plaques

**Fig 1:**
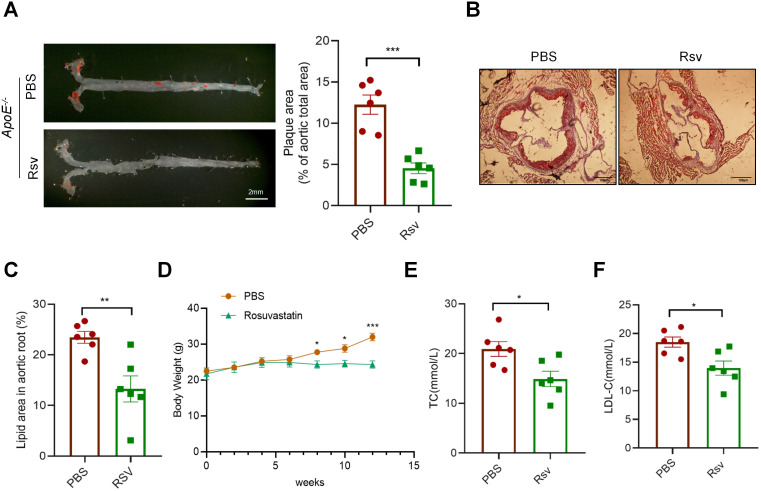
Rosuvastatin inhibits the formation of atherosclerotic plaques in *ApoE*^*-/-*^ mice. (A) Representative en face images of Sudan IV-stained aortas from *ApoE*^*-/-*^ mice treated with PBS or rosuvastatin (5 mg/kg/2 days) followed by a HFD for 12 weeks. The mean aortic lesion area was quantified. Bar = 2 mm. (n ≥ 6 mice per group). ****P < 0.0001 by unpaired Student’s *t* test. (B-C) Representative Oil Red O-stained aortic root sections with quantification of lesion size. Scale bar = 100 μm. **P < 0.01 by unpaired Student’s *t* test. (D) Body weight of each group. *P < 0.05, ***P < 0.001 by unpaired Student’s *t* test. (E-F) Serum TC and LDL-C levels in each group of mice. *P < 0.05 by unpaired Studen*t*’s *t* test.

To investigate the effect of rosuvastatin on atherosclerosis, we gavaged *ApoE*^*-/-*^ mice with rosuvastatin after they were fed a high-fat diet for 6 weeks. Compared with those in the vehicle control group (PBS), the area of aortic plaques in the rosuvastatin treatment group (Rsv) was significantly smaller (Fig 1A). Oil Red O staining also revealed that lipid deposition in the aortic root was markedly lower in the Rsv group than in the PBS group (Fig 1B, C). In addition, the body weights of rosuvastatin-treated mice were significantly lower (Fig 1D), and the low-density lipoprotein and total cholesterol contents were significantly lower (Fig 1E, F), whereas the triglyceride and triglyceride contents were not different (S1A, B Fig). We subsequently conducted nontarget metabolomic analysis on the serum samples of the two groups. The PCA results revealed that the specificity of the two groups was good and that the intragroup differences were small (S1C Fig). Analysis of the differentially abundant metabolites between the two groups revealed that the phosphatidylcholine content in the rosuvastatin treatment group decreased (S1D Fig), and the KEGG pathways of the differentially abundant metabolites were related to cardiovascular diseases (S1E Fig).

### Rosuvastatin prevents the impairment of endothelial cell activity induced by ox-LDL

**Fig 2:**
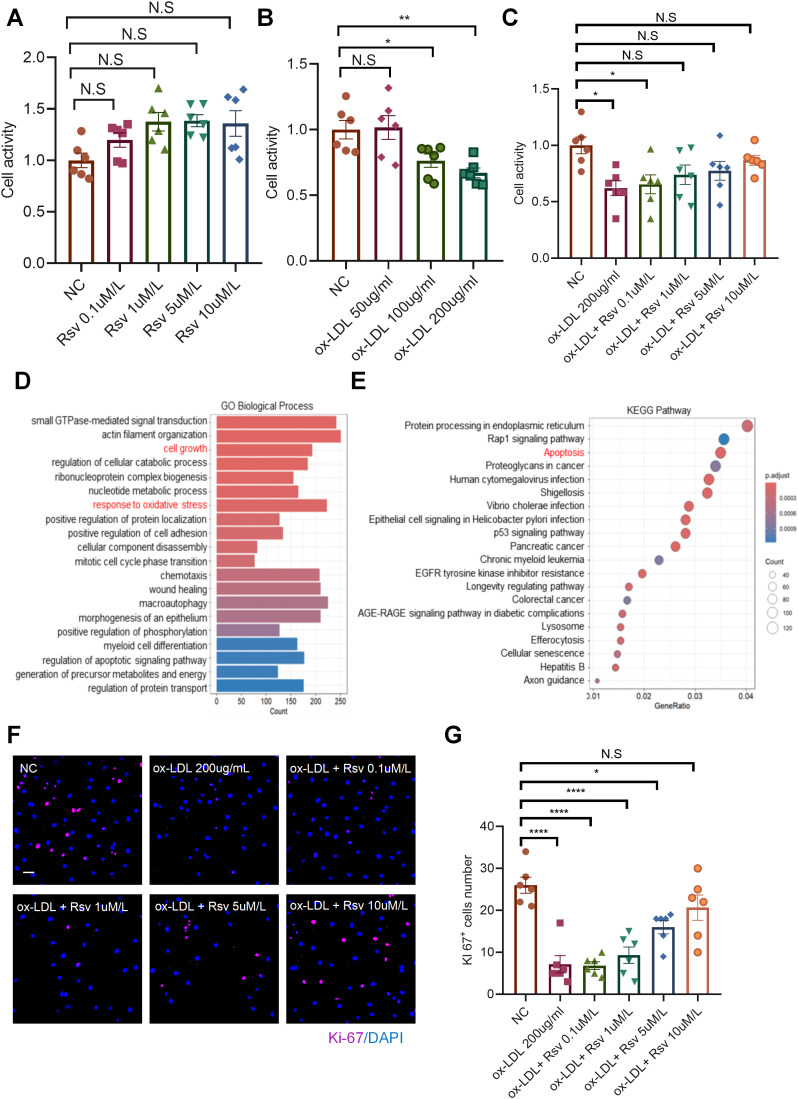
Rosuvastatin prevents the impairment of endothelial cell activity induced by ox-LDL. (A) Effects of different concentrations of rosuvastatin (0.1, 1, 5 and 10 µM) on HUVEC viability for 24 h. (B) Effects of treatment with different concentrations of ox-LDL (50, 100 and 200 µg/mL) for 24 h on HUVEC viability. (C) HUVECs were treated with different concentrations of rosuvastatin and ox-LDL (200 µg/ml) for 24 h. *P < 0.05, ***P < 0.001 by one-way ANOVA. (D) Bar chart showing the signaling pathways enriched with DEGs in the RNA-Seq dataset (GSE206927) of ox-LDL-treated HUVECs according to GO analysis. (E) Bubble chart showing the signaling pathways enriched with DEGs according to KEGG analysis. (F-G) HUVECs stimulated with ox-LDL and different concentrations of rosuvastatin were stained with DAPI (blue) and Ki67 (purple). Ki67-positive cells were quantified, bar = 100 μm, *P < 0.05, ****P < 0.0001 by one-way ANOVA.

Endothelial cell injury is the initial stage of the development of atherosclerosis. Therefore, we aimed to explore the role of rosuvastatin in endothelial cells. To investigate the effect of rosuvastatin on endothelial cell proliferation, we incubated human umbilical vein endothelial cells (HUVECs) with different concentrations of rosuvastatin for 24 h, and cell activity was detected via a CCK8 assay. The results revealed that different concentrations of rosuvastatin had no effect on the viability of endothelial cells (Fig 2A). Circulating LDL crosses the inner membrane and is oxidized to form oxidized LDL (ox-LDL), thereby aggravating damage to the inner membrane. We then incubated HUVECs with different concentrations of ox-LDL for 24 h. CCK8 assay results revealed that the viability of cells stimulated with medium or high concentrations of ox-LDL decreased significantly (Fig 2B), and this phenotype was reversed after coincubation with rosuvastatin (Fig 2C). To explore the effect of ox-LDL on endothelial cells, we analyzed the RNA-Seq data (GSE206927) of human aortic endothelial cells stimulated with ox-LDL. Gene Ontology (GO) analysis revealed that the biological functions of the differentially expressed genes (DEGs) were related mainly to cell growth, the response to oxidative stress, wound healing, etc. (Fig 2D), suggesting that the mechanism of rosuvastatin may be related to its impact on these functions. We also performed Kyoto Encyclopedia of Genes and Genomes (KEGG) analysis, and the results revealed that the DEGs were involved in apoptosis signaling pathways (Fig 2E). To validate the biological processes identified in the transcriptomic GO analysis and investigate the impact of rosuvastatin on ox-LDL-stimulated endothelial cell proliferation, we performed immunofluorescence staining. The results demonstrated that ox-LDL stimulation significantly inhibited cell proliferation compared with that in the control group. Conversely, preincubation with statins markedly reversed the inhibitory effect of ox-LDL on endothelial cell proliferation (Fig 2F and 2G).

### Rosuvastatin inhibits ox-LDL-induced oxidative stress in endothelial cells

**Fig 3:**
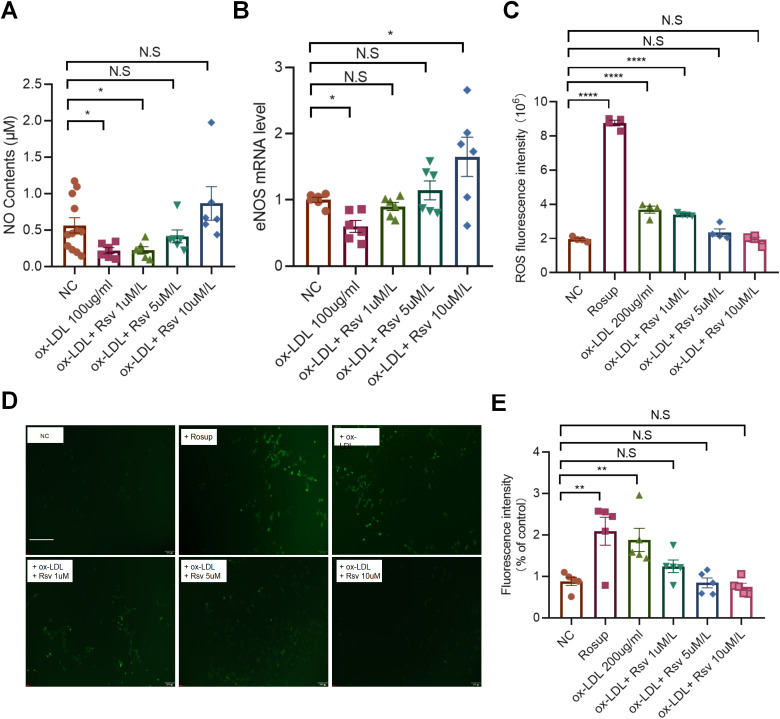
Rosuvastatin inhibits ox-LDL-induced oxidative stress in endothelial cells. HUVECs were treated with ox-LDL in the presence or absence of different concentrations of rosuvastatin (0.1, 1, 5 and 10 µM) for 24 h. (A) NO production in HUVECs. (B) eNOS mRNA expression in HUVECs. (C) A microplate reader was used to measure the fluorescence intensity of the ROS at an excitation wavelength of 488 nm and an absorption wavelength of 525 nm via a fluorescent probe DCFH-DA kit, and Rosup was used as a positive control. (D) The mean intracellular fluorescence intensity was analyzed via fluorescence microscopy. The data are presented as the mean ± SEM. *P < 0.05, **P < 0.01, **P < 0.0001 by one-way ANOVA.

GO analysis suggested that ox-LDL affected the oxidative stress process in endothelial cells. Therefore, we examined the effects of different concentrations of rosuvastatin on the release of nitric oxide (NO) and the expression of nitric oxide synthase (eNOS) in HUVECs induced by ox-LDL. The results revealed a significant increase in NO content after incubation with a low dose of rosuvastatin (Fig 3A), whereas the mRNA expression of eNOS increased significantly after incubation with a high dose of rosuvastatin (Fig 3B). We subsequently detected the production of intracellular reactive oxygen species (ROS) via the use of Rosup as a positive control. After incubation with medium or high concentrations of rosuvastatin, the production of ROS induced by ox-LDL was significantly reversed (Fig 3C). In addition, we directly observed intracellular reactive oxygen species (ROS) fluorescence via fluorescence microscopy, and the results were consistent with the results of the enzyme-labeled fluorescence reading (Fig 3D, E), suggesting that rosuvastatin may protect endothelial cell function by inhibiting ox-LDL-induced oxidative stress.

### Rosuvastatin inhibits ox-LDL-induced apoptosis of endothelial cells

**Fig 4:**
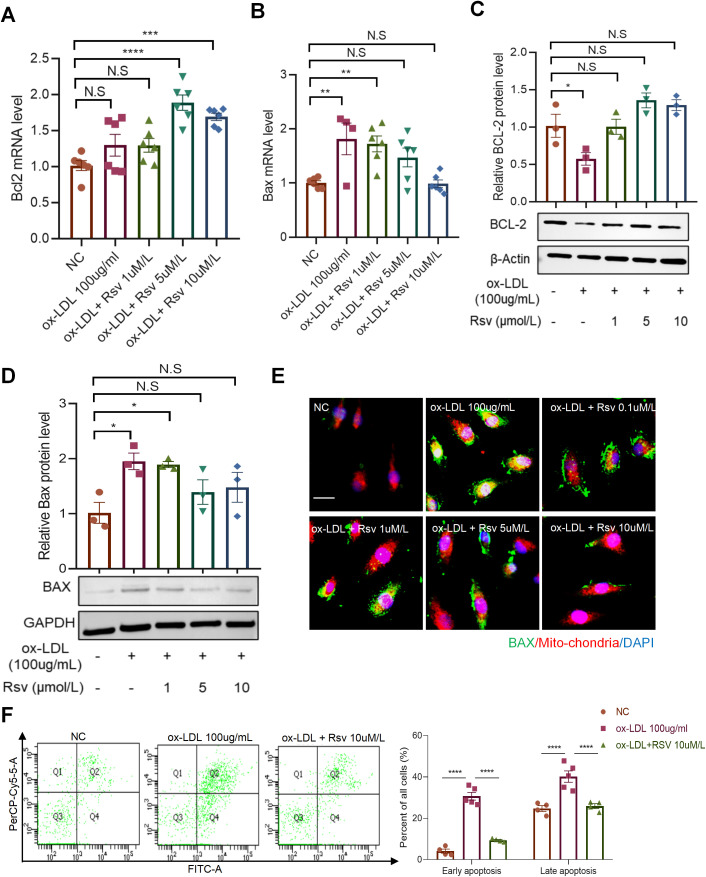
Rosuvastatin inhibits ox-LDL-induced apoptosis of endothelial cells. (A-B) *Bcl2* and *Bax* mRNA expression in HUVECs treated with different concentrations of rosuvastatin and ox-LDL (200 µg/ml) for 24 h. **P < 0.01, ***P < 0.001, ****P < 0.0001 by one-way ANOVA. (C-D) BCL-2 and Bax protein expression in HUVECs treated with different concentrations of rosuvastatin and ox-LDL (200 µg/ml) for 24 h. The data are presented as the means ± SEMs. *P < 0.05 by one-way ANOVA. (E) HUVECs stimulated with ox-LDL and different concentrations of rosuvastatin were stained with DAPI (blue), Bax (green) and mitochondria (red); scale bar = 20 μm. (F) Early and late apoptosis of HUVECs treated with 100 µg/mL ox-LDL and 10 μmol/L rosuvastatin for 24 h. The quantification results are shown on the right (n = 5). ***P < 0.001, ****P < 0.0001 by one-way ANOVA.

KEGG analysis of ox-LDL-stimulated human aortic endothelial cell RNA-Seq data revealed that the DEGs were involved in apoptosis signaling pathways (Fig 2B), indicating that ox-LDL plays an important role in the apoptosis of endothelial cells. We examined the effects of rosuvastatin on the expression of the proapoptotic gene *Bax* and the antiapoptotic gene *Bcl2* in endothelial cells. The results revealed that ox-LDL inhibited Bcl2 protein expression (Fig 4B), and rosuvastatin incubation reversed this phenotype and promoted *Bcl2* mRNA expression (Fig 4A). In addition, rosuvastatin inhibited ox-LDL-induced Bax expression (Fig 4B and 4D). Upon activation, the Bax protein inserts into the outer mitochondrial membrane, leading to a decrease in the mitochondrial membrane potential and subsequent promotion of apoptosis [23]. Therefore, we examined the colocalization of Bax with mitochondria. The results from cellular immunofluorescence showed that after ox-LDL stimulation, a large amount of Bax was expressed in the cytoplasm and mitochondria; however, incubation with high concentrations of rosuvastatin significantly reduced the colocalization of Bax and mitochondria (Fig 4E). These results suggest that rosuvastatin may play a role in protecting endothelial cell function by inhibiting ox-LDL-induced apoptosis. Additionally, we directly assessed the effect of rosuvastatin on apoptosis using flow cytometry, and the results demonstrated that rosuvastatin incubation significantly reversed ox-LDL-induced endothelial apoptosis (Fig 4F).

### Rosuvastatin inhibits the NF-κB signaling pathway in endothelial cells

**Fig 5:**
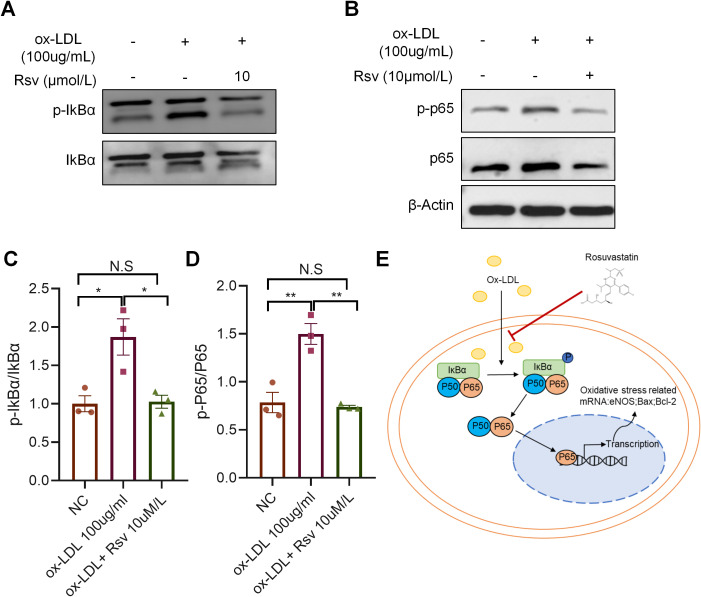
Rosuvastatin inhibits the NF-κB signaling pathway in endothelial cells. (A-D) Protein levels of IkBα, p-IkBα, P65 and p-P65 in HUVECs treated with or without 10 µM rosuvastatin and treated with 100 µg/mL ox-LDL for 24 h. The data are presented as the means ± SEMs. *P < 0.05, **P < 0.01 by one-way ANOVA. (E) Schematic diagram illustrating the role of rosuvastatin in ox-LDL-induced endothelial cell dysfunction. Rosuvastatin regulates oxidative stress and apoptosis-related gene transcription in endothelial cells by inhibiting ox-LDL-induced IKBα and P65 activation in endothelial cells.

NF-κB is a classical inflammatory oxidative stress pathway, and the nuclear translocation of p65 promotes the transcription of related genes. To explore the role of rosuvastatin in protecting endothelial cell function, we used Western blot analysis to detect the expression of pathway molecules in HUVECs stimulated with rosuvastatin and ox-LDL. The results showed that ox-LDL stimulation promoted the phosphorylation of IκBα, while coincubation with rosuvastatin significantly inhibited the activation of IκBα (Fig 5A and 5C). In addition, rosuvastatin reversed the downregulation of P65 expression induced by ox-LDL and inhibited the phosphorylation of P65 (Fig 5B and 5D). These results suggest that rosuvastatin may play a role by inhibiting the NF-κB signaling pathway in ox-LDL-stimulated endothelial cells (Fig 5E).

## Discussion

Endothelial cell dysfunction is a pivotal step in the development of atherosclerosis and inflammation. Dysregulation of lipid metabolism, particularly the oxidation of circulating low-density lipoprotein to form oxidized LDL (ox-LDL), exacerbates endothelial cell injury and atherosclerotic plaque formation. Therefore, pharmacological intervention targeting ox-LDL-induced endothelial dysfunction represents a critical research direction in cardiovascular medicine. In this study, we found that rosuvastatin treatment significantly alleviated plaque formation and reduced blood lipid levels in high-fat diet-induced *ApoE ⁻ / ⁻* mice. Additionally, rosuvastatin may inhibit ox-LDL-induced endothelial cell injury, oxidative stress, and apoptosis by suppressing the NF-κB signaling pathway in HUVECs.

Oxidative stress and apoptosis in endothelial cells accelerate the progression of atherosclerosis through multiple mechanisms. Oxidative stress originates from abnormal expression and activation of NADPH oxidases (NOX1/2/4), mitochondrial electron transport chains, and uncoupled endothelial nitric oxide synthase, leading to excessive production of reactive oxygen species [24]. This triggers the collapse of the mitochondrial membrane potential, induces apoptosis, and disrupts vascular endothelial integrity. Additionally, oxidative stress inhibits endothelial nitric oxide (NO) synthesis and impairs vascular function while enhancing vascular smooth muscle cell proliferation and migration to promote plaque formation [11]. Studies have shown that endothelial oxidative stress regulates apoptosis and inflammatory responses by activating the NF-κB signaling pathway [25]. ROS directly induce the phosphorylation of IκB kinase (IKK), leading to the degradation of the inhibitory protein IκB and the release of NF-κB dimers into the nucleus. This upregulates the expression of the adhesion molecules VCAM-1 and ICAM-1 [26]. Moreover, NF-κB disrupts the Bcl-2/Bax balance by inhibiting the transcription of the antiapoptotic protein Bcl-2, exacerbating the activation of the mitochondrial apoptosis pathway [27]. In ox-LDL-induced endothelial injury, NF-κB activation and the NO/eNOS pathway are reciprocally inhibited. Oxidative stress causes eNOS uncoupling, reducing NO production, whereas decreased NO promotes NF-κB nuclear translocation through S-nitrosylation, forming a vicious cycle. Furthermore, NF-κB directly regulates the expression of NADPH oxidase subunits (such as p47phox), enhancing ROS production and reinforcing oxidative stress signals [28–30]. This interaction promotes endothelial inflammation and apoptosis in the early stage of atherosclerosis and accelerates lesion progression by facilitating foam cell formation and plaque instability in the late stage. Our results are consistent with those of previous studies; after ox-LDL stimulation of HUVECs, endothelial NO and eNOS expression significantly decreases, ROS expression markedly increases, and the bax/bcl2 ratio significantly increases. Mechanistically, cellular IκBα and p65 phosphorylation are significantly increased, indicating activation of the NF-κB pathway. Apoptotic endothelial cells also release damage-associated molecular patterns (DAMPs, such as HMGB1), which activate macrophages via TLR4 to secrete proinflammatory cytokines such as IL-1β and TNF-α, forming a positive “oxidative‒inflammatory” feedback loop [27]. Although our study confirmed that ox-LDL can induce endothelial oxidative stress and apoptosis through NF-κB activation, several questions require further investigation: do other members of the NF-κB family, such as P52/RELB, play a role? Can rosuvastatin targeting NF-κB reduce ox-LDL-induced endothelial inflammation? These areas warrant further exploration.

Rosuvastatin, a highly effective statin, reduces circulating cholesterol levels by limiting the production of cholesterol precursors and promoting the catabolism of LDL, a process achieved by increasing the expression of LDL receptors on hepatocyte surfaces. Relevant studies have shown that both atorvastatin and rosuvastatin can downregulate the expression of pro-inflammatory cytokines such as IL-1β in HUVECs, but rosuvastatin has the strongest anti-inflammatory activity and the most potent effect on mitigating oxysterol-induced impairment [31]. Among statin medications, rosuvastatin has been extensively investigated owing to its robust lipid-lowering efficacy and low adverse event risk [32]. At the cellular level, rosuvastatin has positive effects on the functions of vascular cells. Studies have shown that rosuvastatin can significantly improve endothelial cell function, inhibit endothelial inflammatory responses, suppress the activation of nuclear factor kappa-B, and reduce the release of IL-1β and TNF-α [33]. Additionally, rosuvastatin inhibits ox-LDL-induced endothelial dysfunction, promotes the expression of eNOS, and enhances its phosphorylation at the Ser177 site, thereby increasing the bioavailability of NO [10]. Moreover, rosuvastatin reduces macrophage uptake of ox-LDL and decreases the formation of foam cells [34]. Furthermore, rosuvastatin inhibits the proliferation of smooth muscle cells by blocking the hydroxymethylglutaryl acid pathway through inhibiting the formation of isoprenoid metabolites, which helps stabilize atheromatous plaques and slows lesion progression [35]. Similar to other studies, we pretreated ox-LDL-stimulated HUVECs with 1, 5, and 10 μmol/L rosuvastatin and found that cell activity increased with increasing rosuvastatin concentration, accompanied by significantly increased NO production and eNOS expression. Notably, rosuvastatin also reduced intracellular ROS production and cellular oxidative stress in a dose-dependent manner. These results further confirm the protective effects of rosuvastatin on endothelial cells. KEGG analysis revealed that the DEGs were enriched in the apoptosis signaling pathway, and we confirmed this pathway through flow cytometry analysis and detection of the expression of the apoptosis-related molecules BAX and BCL-2. Future research could employ global transcriptome sequencing and gene editing techniques in rosuvastatin-pretreated endothelial cells to explore the transcription factors and signaling pathways through which rosuvastatin regulates genes associated with endothelial oxidative stress and apoptosis. Furthermore, DEGs in ox-LDL-stimulated endothelial cells were enriched in the p53 signaling pathway, which is a transcription factor in a key pathway that governs apoptosis and autophagy. Whether statins affect endothelial cell function through the p53 pathway warrants further investigation in subsequent studies. Furthermore, differentially expressed genes (DEGs) in ox-LDL-stimulated endothelial cells were enriched in the p53 signaling pathway. As a key regulatory pathway that intersects with both apoptosis and autophagy, the p53 signaling pathway plays a critical role in governing cell survival and death. We further examined the effects of ox-LDL and rosuvastatin on autophagy in endothelial cells. The results revealed that rosuvastatin treatment reversed ox-LDL-induced autophagic flux in endothelial cells, reduced the protein expression of LC3Ⅰ/Ⅱ, and increased the protein expression of the substrate P62 (S2 Fig). However, the specific mechanism by which it interacts with the NF-κB pathway, requires further verification. Although we confirmed in vitro that rosuvastatin can directly inhibit the NF-κB pathway and oxidative stress in ox-LDL-stimulated HUVECs, indicating that statins exert a direct vascular protective effect that is independent of their lipid-lowering effect. Notably, its lipid-lowering property itself may also be conducive to protecting endothelial function, and these two effects may synergistically mitigate atherosclerosis. Another limitation of this study is that we investigated the effect of rosuvastatin only in male *ApoE*^*-/-*^ mice and did not explore its role in female mice. Whether estrogen influences the efficacy of statins in the treatment of cardiovascular diseases warrants further exploration.

In summary, we demonstrated that rosuvastatin reverses ox-LDL-induced endothelial cell injury and oxidative stress by inhibiting the activation of the NF-κB signaling pathway, promoting the expression of endothelial nitric oxide and endothelial nitric oxide synthase, and inhibiting endothelial apoptosis. Our findings indicate that rosuvastatin protects endothelial cell function via the stimulation of ox-LDL and the progression of atherosclerosis, providing a theoretical basis for the application of statin drugs in the prevention and treatment of diseases caused by endothelial cell injury, such as atherosclerosis.

## Supporting information

S1 Fig Effects of rosuvastatin on the metabolism of *ApoE*^*-/-*^ mice. (A-B) Serum TG and HDL-C levels in each group of mice. (C) PCA analysis of the serum from *ApoE*^*-/-*^ mice treated with PBS or rosuvastatin. (n = 6 mice per group). (D) Heatmap analysis of differentially abundant metabolites. (E) Histogram of the results of the KEGG analysis of differentially abundant metabolites.
(TIF)

S2 Fig Rosuvastatin inhibits ox-LDL-induced autophagy in endothelial cells. (A-C) LC3Ⅰ/Ⅱ and P62 were detected and quantified in HUVECs stimulated by ox-LDL for 24 h with or without rosuvastatin (10 μmol/L). *P < 0.05 by one-way ANOVA, n = 3 per group.
(TIF)

S3 Fig Full western blots for panels Figs 4C, 4D, 5A, 5B and S2A. All with the corresponding control. Blots were cut at the position of the box.
(TIF)

S1 Table List of KEGG-Metabset Enrich in this study. (XLSX)

S1 Raw Images (PDF)
