# Supplementary figures and images for: Rosuvastatin protects against oxLDL-induced endothelial cell oxidative stress and attenuates atherosclerotic plaque formation in ApoE-/- mice through the NF-κB pathway
Source: PLoS One. 2026 Feb 20;21(2):e0339967. doi: 10.1371/journal.pone.0339967 (PMC12923013; doi:10.1371/journal.pone.0339967)

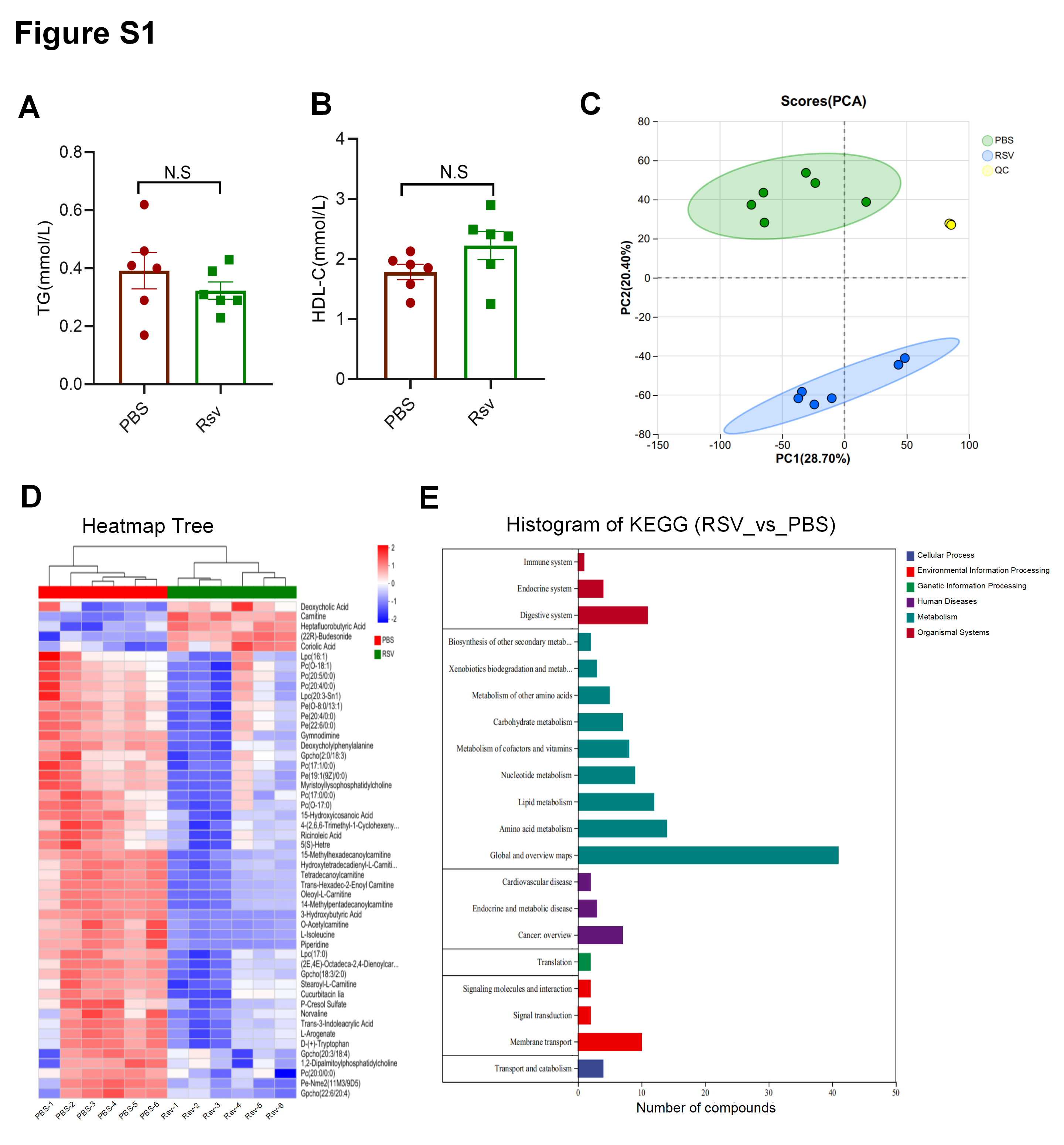

Supplement: S1 Fig — (A-B) Serum TG and HDL-C levels in each group of mice. (C) PCA analysis of the serum from ApoE-/- mice treated with PBS or rosuvastatin. (n = 6 mice per group). (D) Heatmap analysis of differentially abundant metabolites. (E) Histogram of the results of the KEGG analysis of differentially abundant metabolites. (TIF) [file pone.0339967.s001.tif]

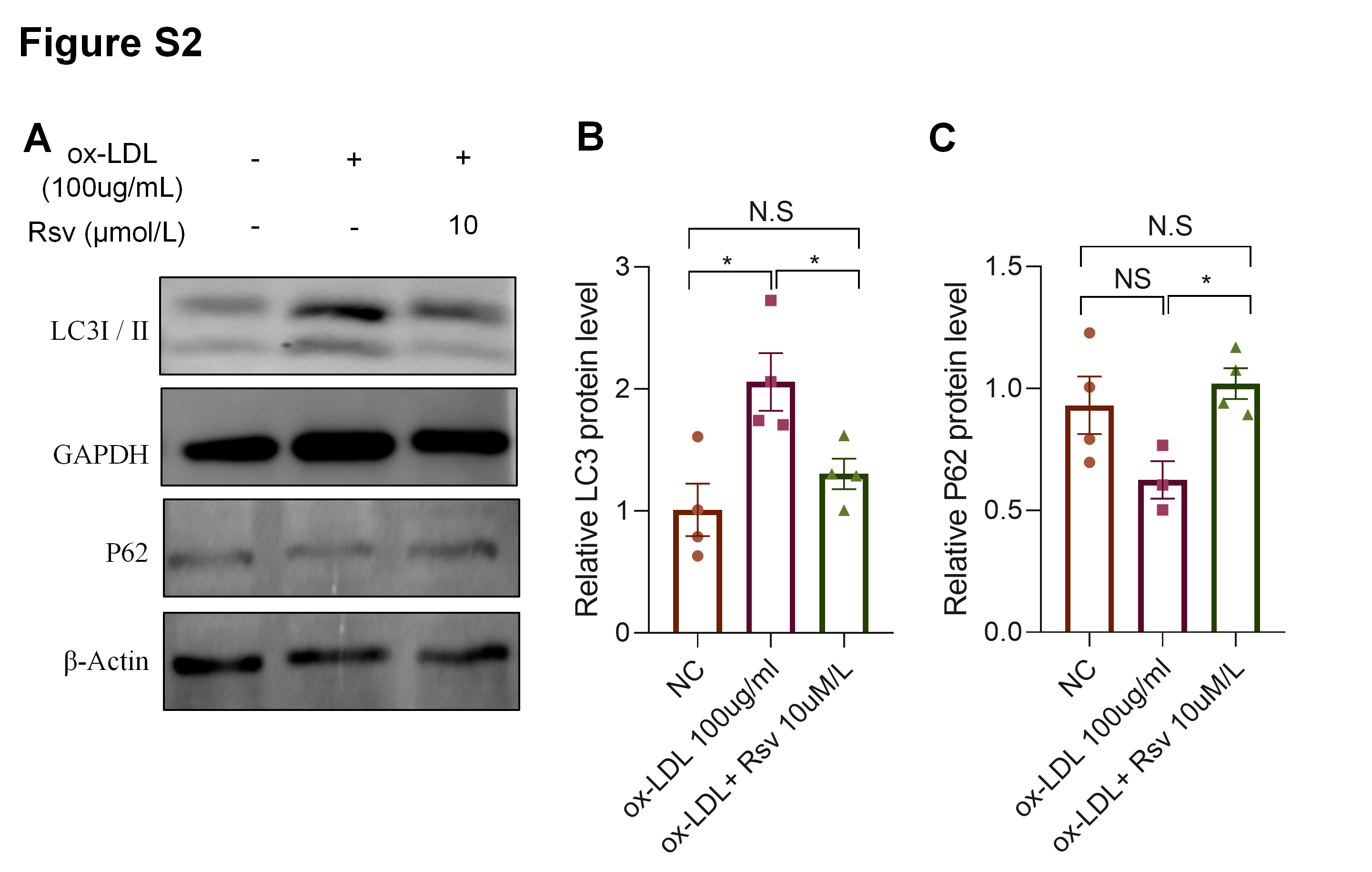

Supplement: S2 Fig — (A-C) LC3Ⅰ/Ⅱ and P62 were detected and quantified in HUVECs stimulated by ox-LDL for 24 h with or without rosuvastatin (10 μmol/L). *P < 0.05 by one-way ANOVA, n = 3 per group. (TIF) [file pone.0339967.s002.tif]

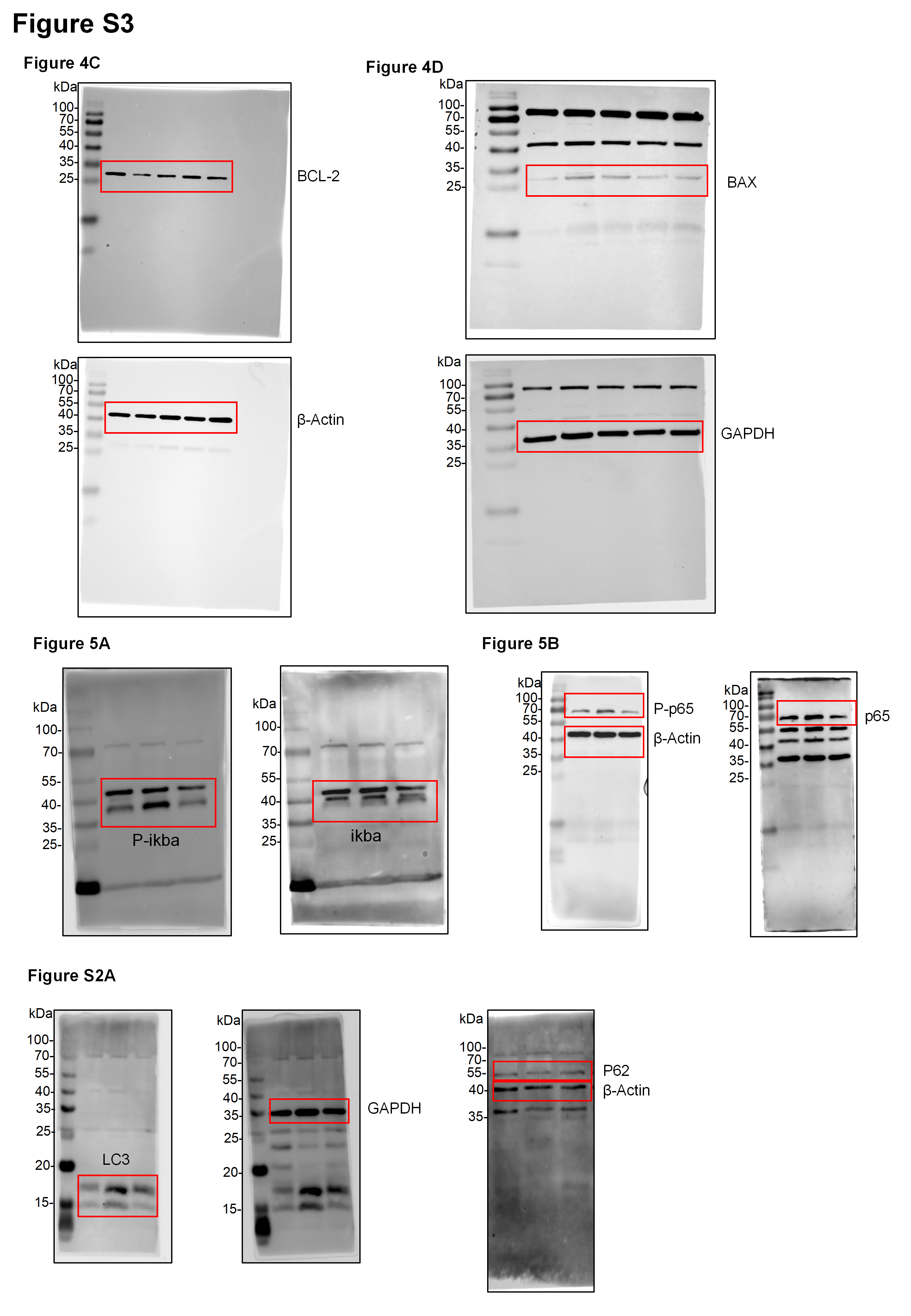

Supplement: S3 Fig — All with the corresponding control. Blots were cut at the position of the box. (TIF) [file pone.0339967.s003.tif]
