# Supplementary material for: Rosuvastatin protects against oxLDL-induced endothelial cell oxidative stress and attenuates atherosclerotic plaque formation in ApoE-/- mice through the NF-κB pathway
Source: PLoS One. 2026 Feb 20;21(2):e0339967. doi: 10.1371/journal.pone.0339967 (PMC12923013; doi:10.1371/journal.pone.0339967)

Supp 1. Original blot images and statistic analysis for Figure 4C

A

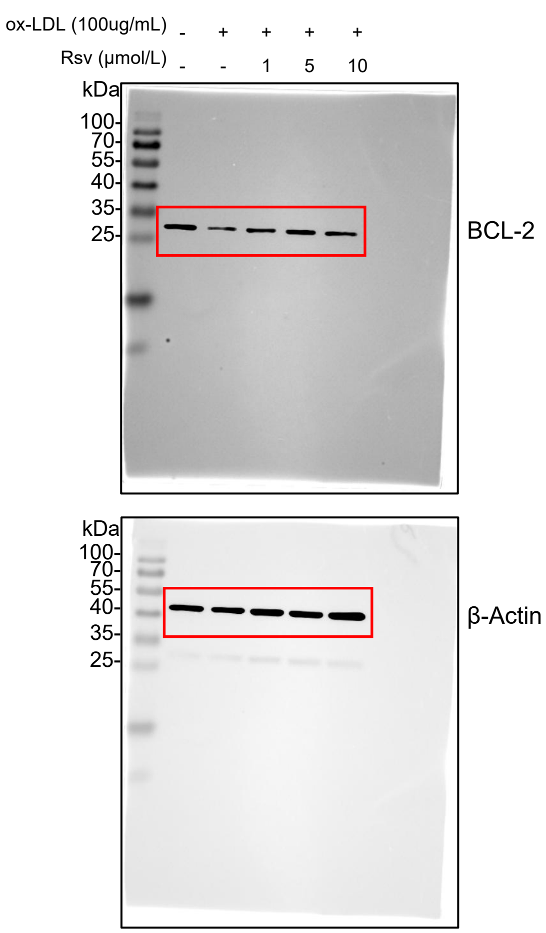

B

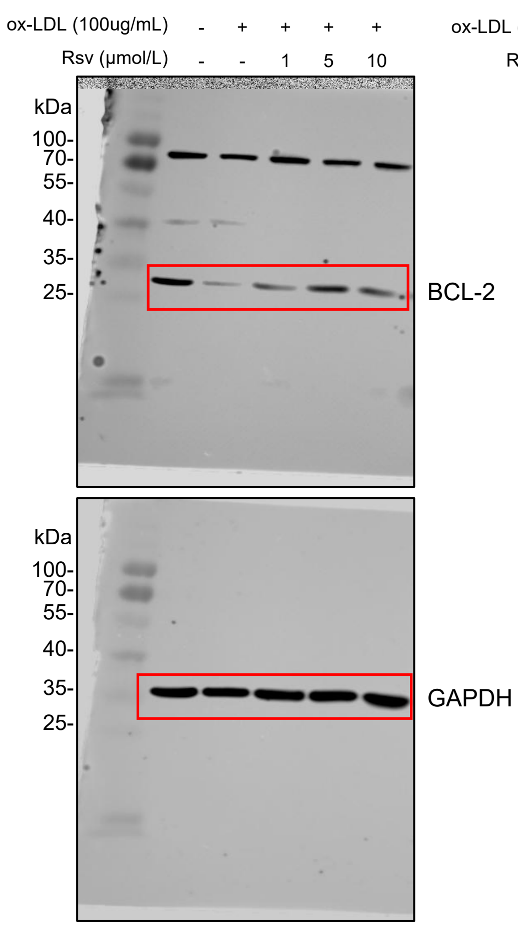

C

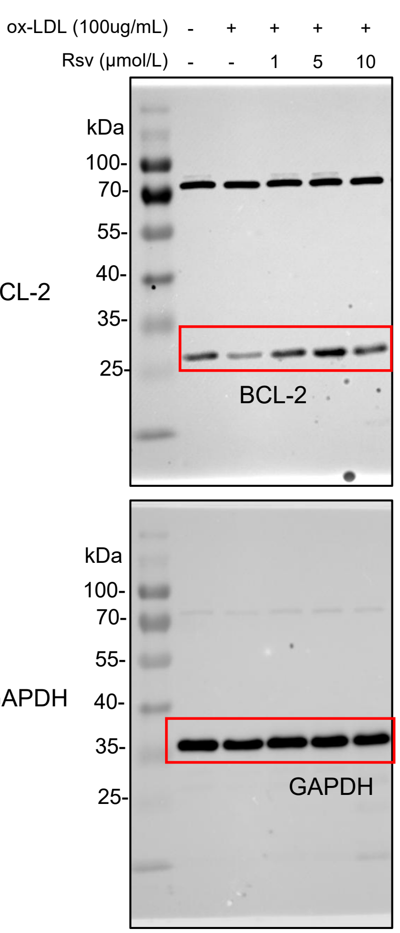

D

①						mean				
90096	50418	74620	115035	113043		0.555065	0.387673	0.463113	0.760889	0.602593
162316	130052	161127	151185	187594		0.685008	0.224622	0.450269	0.674951	0.54403
0.555065	0.387673	0.463113	0.760889	0.602593		0.408893	0.316118	0.625375	0.810974	0.735836
						0.549656				
②										
149922	51355	104779	163086	150522						
218862	228629	232704	241626	276679						
0.685008	0.224622	0.450269	0.674951	0.54403						
③						normalize				
117619	81885	179798	221565	196728		1.027899	0.717912	0.857617	1.409054	1.115913
287652	259033	287504	273208	267353		1.268533	0.415967	0.833831	1.24991	1.007463
0.408893	0.316118	0.625375	0.810974	0.735836		0.75721	0.585403	1.158102	1.501804	1.362659

E

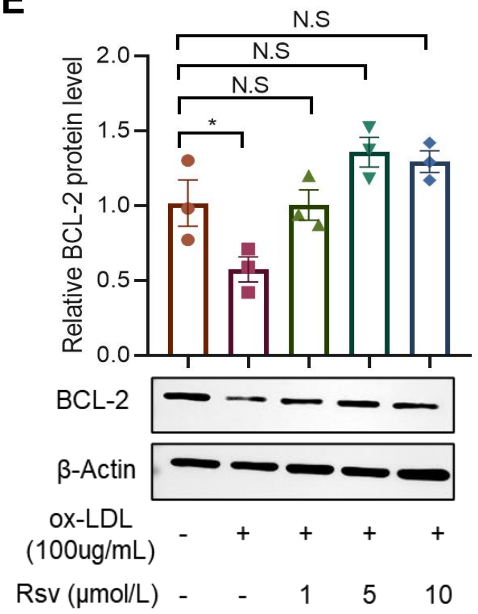

Supp 2. Original blot images and statistic analysis for Figure 4D

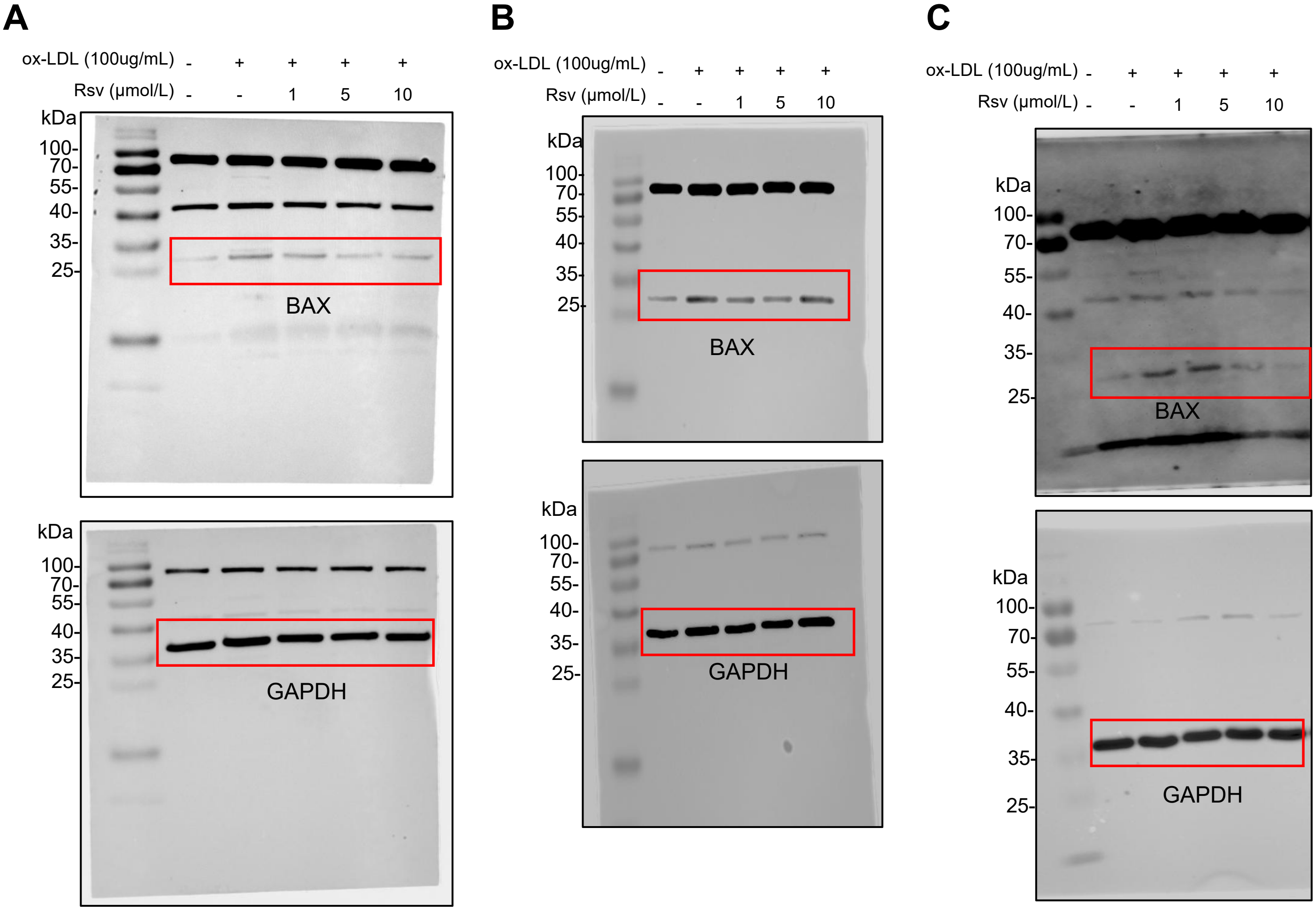

D

①						mean				
24816	69727	57480	40421	49318		0.09268	0.22769	0.2187	0.15975	0.19468
267758	306235	262828	253026	253325		0.1669	0.26824	0.22296	0.21677	0.22337
0.09268	0.22769	0.2187	0.15975	0.19468		0.10608	0.20681	0.24035	0.12508	0.11452
						0.12189				
②										
24205	56242	35077	33670	50129						
145026	209670	157322	155324	224424						
0.1669	0.26824	0.22296	0.21677	0.22337						
③						normalize				
34585	72869	72222	40527	34510		0.77234	1.89743	1.82248	1.33125	1.62236
326027	352351	300490	324021	301350		1.39084	2.23534	1.85803	1.80644	1.86139
0.10608	0.20681	0.24035	0.12508	0.11452		0.884	1.7234	2.0029	1.04229	0.95432

E

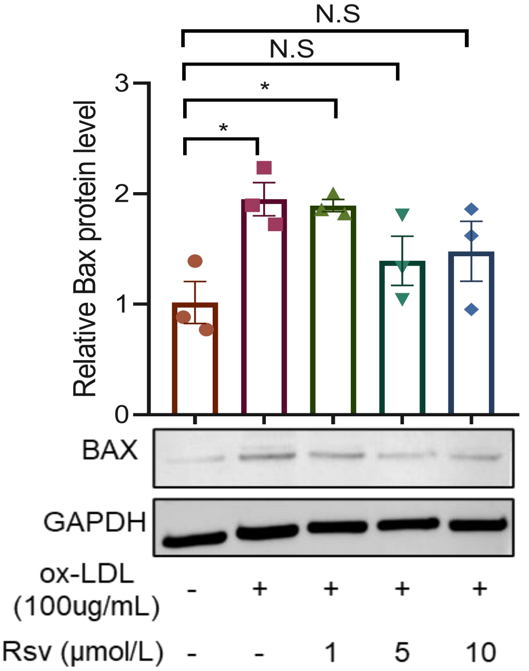

Supp 3. Original blot images and statistic analysis for Figure 5A

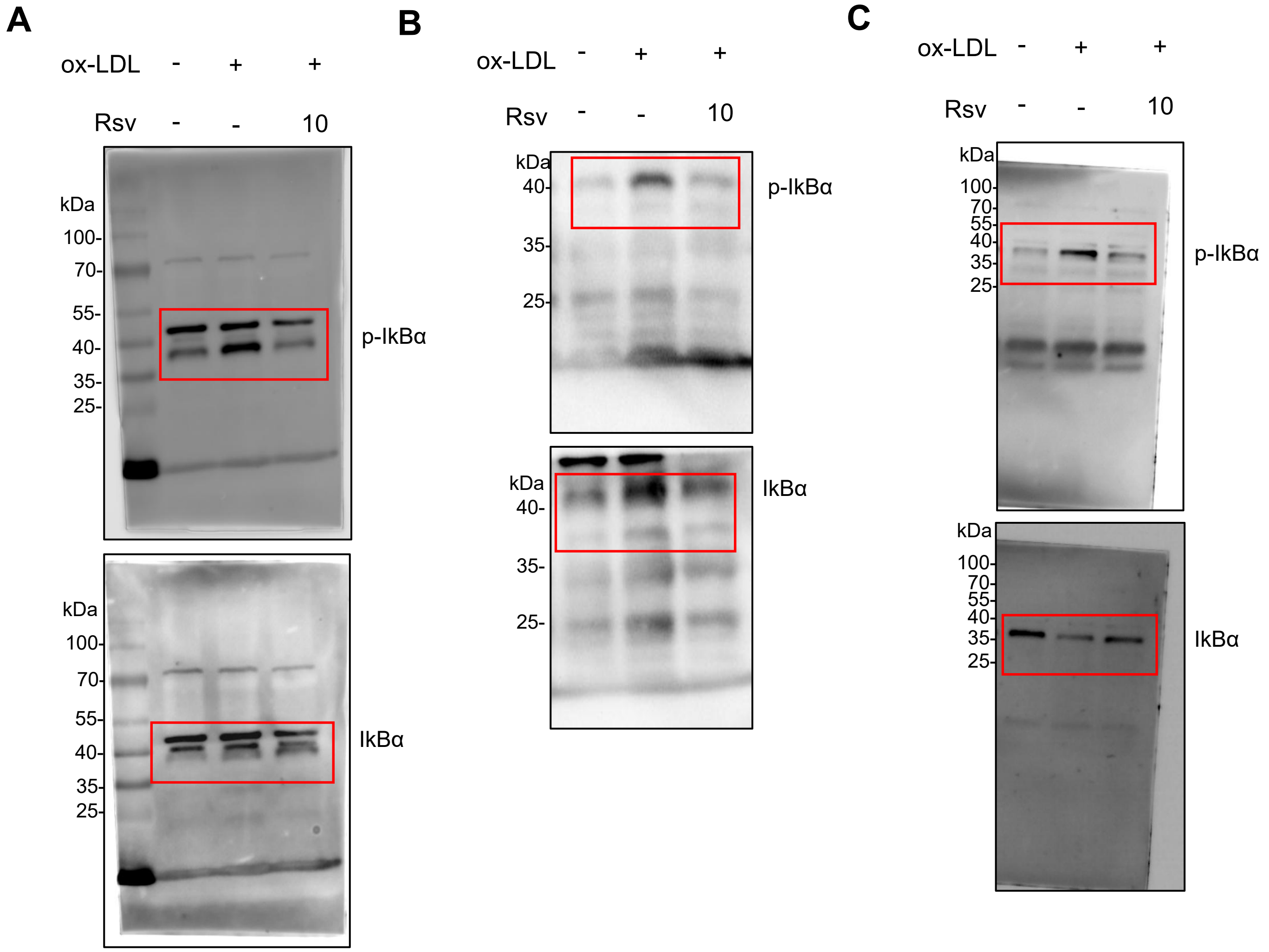

D

①					mean		
299849	566780	284584			0.83895	1.37178	0.80523
357410	413171	353419			0.61848	0.98378	0.60474
0.83895	1.37178	0.80523			0.62683	1.53884	0.72886
					0.69476		
②							
151024	300071	209677			normaliz		
93406	295203	126801			1.20886	1.97663	1.16028
0.61848	0.98378	0.60474			0.89119	1.41755	0.87139
					0.90322	2.21735	1.05024
③							
63839	135647	72627					
101844	88149	99644					
0.62683	1.53884	0.72886					

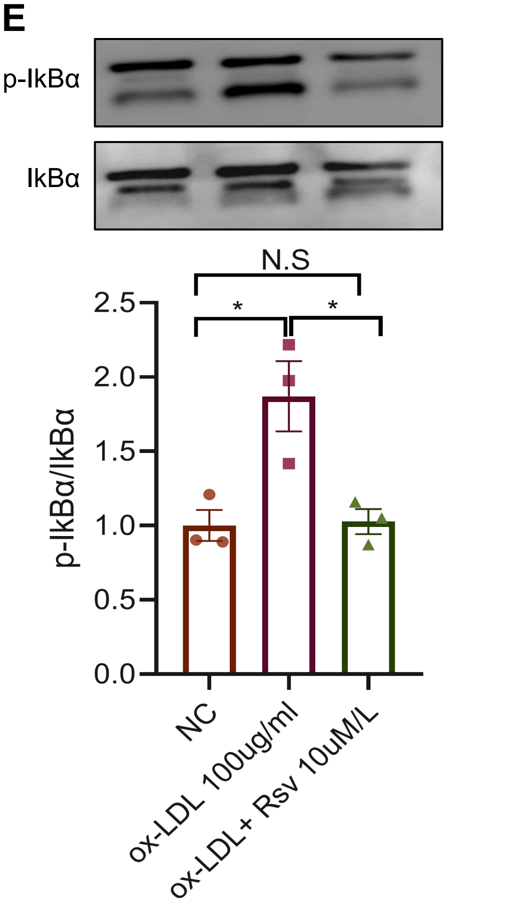

Supp 4. Original blot images and statistic analysis for Figure 5B

A

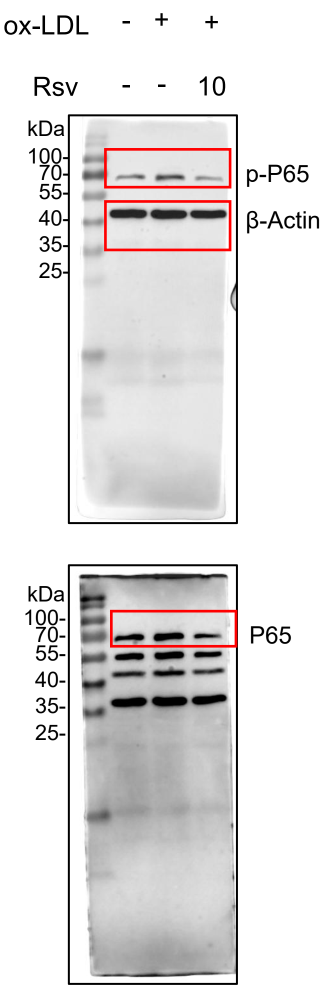

B

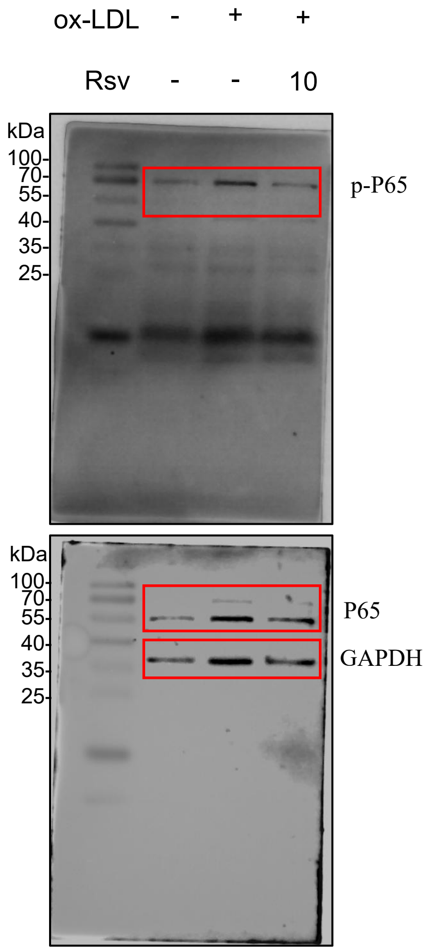

C

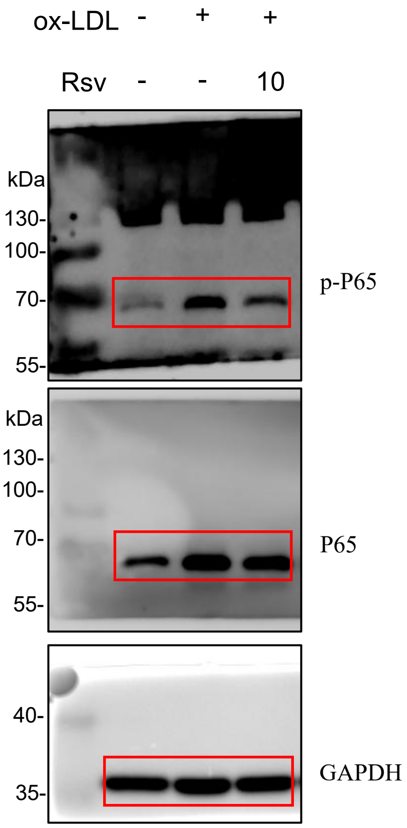

D

①				mean		
34626	69950	29337		0.274307	0.571034	0.2781
126231	122497	105491		0.383799	0.654411	0.298559
0.274307	0.571034	0.2781		0.251079	0.510912	0.279615
				0.303062		
②				normalize		
25424	59147	25774		0.710639	1.479364	0.720465
66243	90382	86328		0.994298	1.695366	0.773469
0.383799	0.654411	0.298559		0.650464	1.323606	0.72439
③						
99577	280366	133450				
396596	548756	477264				
0.251079	0.510912	0.279615				

E

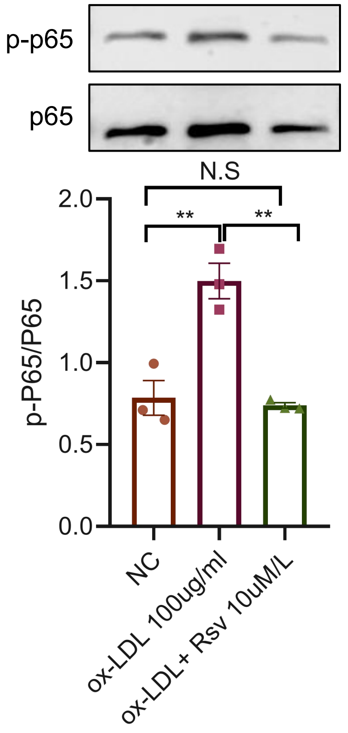

Supplement: S1 Raw Images — (PDF) [file pone.0339967.s005.pdf]
